# Open tools for quantitative anonymization of tabular phenotype data: literature review

**DOI:** 10.1093/bib/bbac440

**Published:** 2022-10-10

**Authors:** Anna C Haber, Ulrich Sax, Fabian Prasser, the NFDI4Health Consortium

**Affiliations:** Health Data Science Center, Berlin Institute of Health at Charité - Universitätsmedizin Berlin, Berlin, Germany; Department of Medical Informatics, University Medical Center Göttingen, Göttingen, Germany; Campus-Institute Data Science, Georg-August-University Göttingen; Health Data Science Center, Berlin Institute of Health at Charité - Universitätsmedizin Berlin, Berlin, Germany

**Keywords:** privacy, phenotype, data anonymization, software, review

## Abstract

Precision medicine relies on molecular and systems biology methods as well as bidirectional association studies of phenotypes and (high-throughput) genomic data. However, the integrated use of such data often faces obstacles, especially in regards to data protection. An important prerequisite for research data processing is usually informed consent. But collecting consent is not always feasible, in particular when data are to be analyzed retrospectively. For phenotype data, anonymization, i.e. the altering of data in such a way that individuals cannot be identified, can provide an alternative. Several re-identification attacks have shown that this is a complex task and that simply removing directly identifying attributes such as names is usually not enough. More formal approaches are needed that use mathematical models to quantify risks and guide their reduction. Due to the complexity of these techniques, it is challenging and not advisable to implement them from scratch. Open software libraries and tools can provide a robust alternative. However, also the range of available anonymization tools is heterogeneous and obtaining an overview of their strengths and weaknesses is difficult due to the complexity of the problem space. We therefore performed a systematic review of open anonymization tools for structured phenotype data described in the literature between 1990 and 2021. Through a two-step eligibility assessment process, we selected 13 tools for an in-depth analysis. By comparing the supported anonymization techniques and further aspects, such as maturity, we derive recommendations for tools to use for anonymizing phenotype datasets with different properties.

## INTRODUCTION

### Background

Complex diseases are caused by a combination of genetic and environmental factors. Determining their cause, optimal therapies and prognosis requires matching clinical phenotypes with underlying biomolecular mechanisms [1]. Using molecular and systems biology methods as well as bidirectional association studies of phenotypes and (high-throughput) genomic data, researchers have made significant progress in many areas, including oncology [2]. Research on such precision medicine approaches is funded through large-scale initiatives in many countries, including the USA [3] and China [4].

However, the integrated use of such data often faces obstacles and leads to legal and ethical issues, especially in regards to data protection [5]. There is also a strong push towards making research data more i.e. Findable, Accessible, Interoperable and Reusable (FAIR) [6], which also requires addressing privacy challenges when data are processed for secondary purposes or disclosed to third parties [7]. To process data in compliance with regulations, organizational and legal procedures need to be implemented to protect the privacy of patients and research participants (see e.g. [8, 9]). An important prerequisite for data processing in medical research is usually informed consent [10]. However, collecting consent can be difficult and is not always feasible, in particular when data are to be analyzed retrospectively and at large scale [11].

An alternative approach for phenotype descriptions is a process in which data are altered in such a way that the risk of re-identification of individual patients and research participants is minimized. This protection mechanism is called de-identification in some jurisdictions, such as the USA [8], and anonymization (the convention we use in this paper) in others, e.g. the European Union [9]. We also note that the term anonymization can be understood in a legal sense, which means that re-identification risks are reduced so far that the data can be considered non-personal and regulations do not apply anymore, as well as in a technical sense, where it merely means that some anonymization operations have been applied.

From the methodological perspective, anonymization means to transform data in such a way that privacy risks are reduced, while the reduction in risks is balanced against a reduction in the utility of the data. Several high-profile re-identification attacks have shown that achieving low risks can be complex [12]. For example, simply removing directly identifying attributes such as names or personal identifiers is usually not enough to prevent re-identification. More formal approaches are needed that use mathematical and statistical models to quantify risks and guide their reduction. Three different general types of models and methods are usually required for this purpose [13]:

(i) Privacy models implement techniques for quantifying risks to the privacy of individuals. Well-known examples include k-anonymity [14], which captures the uniqueness of combinations of key variables that could be used for re-identifying individuals, but there are further approaches, e.g. based on statistical modelling [15], game-theory [16] or differential privacy [17].
(ii) Transformation models implement techniques for modifying data to reduce risks according to the privacy models supported. Typical examples include generalization, deletion of data [18] or the addition of noise.
(iii) Utility models are used for quantifying the usefulness of output data to guide the anonymization processes. Often, aspects such as data quality or loss of information are used as proxies. A distinction can be made between general-purpose methods reflecting data fidelity or changes to value distributions as well as application-specific models, e.g. for creating privacy-preserving machine learning models [18].

We note that anonymization approaches have traditionally been developed in the statistics as well as the computer science community. The former community focused more on informal and ‘rules-of-thumb’ approaches aiming towards utility preservation, which were, e.g. used to publish census data, while the latter focused on more rigorous algorithmic approaches providing formal protection guarantees motivated by successful privacy breaches. In recent years, the approaches taken by both communities have started to converge [19]. Moreover, we would like to point out that algorithmic approaches to tabular data anonymization are ‘white box’ approaches, meaning that is possible to clearly understand why a certain result has been produced for a certain dataset provided a certain configuration. However, anonymization can still lead to biases in data, e.g. when outliers are removed. For this reason, every anonymization process needs to be guided by a thorough human-in-the-loop utility analysis, which can be supported by the models introduced above and the tools reviewed in this article.

By configuring and applying anonymization tools according to the requirements laid out in laws, regulations and guidelines they can be used achieve anonymity in the legal sense. Examples include the Safe Harbor and Expert Determination methods defined by the US Health Insurance Portability and Accountability Act (HIPAA) Privacy Rule [8]^,^ quantitative anonymization approaches oriented towards the EU General Data Protection Regulation [9] as well as the methods laid out in the UK National Health Service Digital (NHS Digital) Standard for Publishing Health Data [20]. Methods for anonymization are relevant on a global scale, e.g. also in China, which just recently enacted its new Personal Information Protection Law (PIPL) [21]. Moreover, by utilizing anonymization on a best effort basis, risks can be reduced and best practices, such as the data minimization principle, can be implemented.

### Objective

When aiming to integrate anonymization methods into their data processing pipelines, researchers and data scientists are confronted with a range of challenges. First, a very large number of anonymization techniques have been proposed in the literature [22]. Moreover, implementing them in a scalable and reliable manner is difficult [23]. Common libraries and mature anonymization tools provide a viable alternative by implementing a carefully selected set of methods in a reliable manner. However, also the space of available anonymization tools is heterogeneous and obtaining an overview of their strengths and weaknesses is difficult due to the complexity of the problem space and the wide range of implementation-specific properties.

To support researchers with putting anonymization procedures into practice, we hence performed a systematic review of anonymization tools described in the literature. We focused on software for structured tabular data, as this is the most prevalent type of structure for phenotype descriptions. For an overview of anonymization approaches for other data types, we refer to the ‘Discussion’ section. We further focused on open tools, mostly from academic institutions, as little is known about the design and inner workings of commercial software in the space, which is not readily available to the scientific community. The major goals of our work were to provide a systematic overview of anonymization tools for structured data available to the community by answering the following research questions:

(i) Which open tools for anonymizing structured tabular data have been described in the literature?
(ii) What is their technological basis, maturity and development status?
(iii) What are the tools’ advantages and disadvantages?

We believe that this information is vital for enabling researchers choose the right tools for their use cases.

## METHODS

We performed a structured review to map the state-of-the-art in the broad and heterogeneous field of anonymization tools for tabular data. Where applicable, we followed the Preferred Reporting Items for Systematic Reviews and Meta-Analyses (PRISMA) guidelines for structured reviews [24]. After a structured selection process, we created summarized descriptions of the different tools, charted data items describing their development status and functionality and then compared the data items to answer the research questions outlined above. Because the data were obtained from published papers and software solutions, ethical approval was not sought for this study.

### Selection process

**Figure 1:**
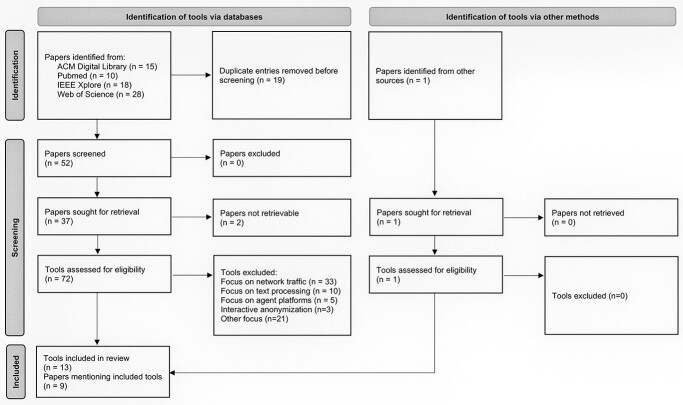
Flow diagram of the selection process for anonymization tools (based on [24]).

Our aim was to identify open anonymization tools for tabular data that have been described in the scientific literature. An overview of the screening and selection process is shown in Figure 1. The complete selection process is described in Supplement 1.

We searched PubMed, IEEE Xplore, the ACM Digital Library and the Web of Science, as the topic of this review is placed at the intersection of medicine and computer science. We used two sets of keywords, one to cover the anonymization context and one to cover the software context. The final search was performed on 22 July 2022. Eligible articles needed to match both sets in their title:

(i) Term set 1: ‘anonymization’ or ‘anonymization’ or ‘anonymizing’ or ‘anonymizing’ or ‘anonymous’ or ‘anonymity’.
(ii) Term set 2: ‘tool’ or ‘software’.

We further restricted the results to peer-reviewed articles, written in English and describing original work published between 1990 and 2021. We chose this timeframe to ensure that we include all relevant literature. Research on anonymization received a significant impetus with the re-identification of Massachusetts Governor William Weld’s medical records in 1997 [12, 14]. The search for studies published up to seven years prior to this event was intended to ensure that no earlier developments are missed.

Search results were exported as comma-separated values files, harmonized, and imported into a consolidated spreadsheet. In total, we found 52 articles (without duplicates), of which 15 were identified via the ACM Digital Library, 10 via PubMed, 18 via IEEE Xplore and 28 through the Web of Science (including duplicates). We added one additional article covering an anonymization tool that we found through the website of the organization Technology, Methods, and Infrastructure for Networked Medical Research (TMF), which develops best practices for networked medical research in Germany [25]. From the articles identified, we extracted tools that were mentioned as well as their application scope. Only tools for anonymizing structured tabular data were considered for further analysis. Each paper was screened by one of the authors AH or FP and the results were checked by the corresponding other author. Cases of disagreement were discussed until consensus was reached. The largest number of tools was excluded due to a focus on network traffic anonymization (32), text processing (10), agent platforms (5) and interactive query anonymization (3). In total, 13 tools were identified as being eligible for in-depth analysis.

### Data charting

For the eligible tools, we collected basic properties such as the programming language utilized, the date of the first release and last known update as well as information on the functionalities supported. Data charting was done by AH and FP following the same process as in the screening step. An overview of the set of data items charted is provided in Table 1.

**Table 1:** Data items collected

Variable	Examples	Definition
Institution	Statistics Austria, University of Thessaly	Institution of the primary author of the software
Country	Austria, Germany	Country in which the institution of the primary author of the software is located
Language	Java, R	Programming language in which the software is primarily implemented
Release date	2012	Year in which the software was first released
Latest update	2019	Year in which the latest update or changes to the software were performed
License	MIT license	License under which the source of the software is released
Privacy models	k-anonymity, l-diversity	Models supported by the software for measuring and automatically reducing privacy risks

We note that we were particularly careful along the functionality axis. It is challenging to compare different anonymization tools to each other without comparing apples to oranges (a problem that often occurs in sections on related work of corresponding papers). For example, several tools might support a certain privacy model—but only in combination with different degrees of automation and different ways of transforming data. Moreover, a certain anonymization method may only be supported while optimizing output data towards a specific objective, such as maximizing output data fidelity, which might not result in data that is suitable for other use cases, such as machine learning applications [26]. To obtain comparable data items, we hence focused only on privacy models that can be enforced by the tools in an automated manner. This also provides hints to whether a certain tool is suitable for generic biomedical research data platforms where a high degree of automation is often needed.

## Results

In this section, we will first present an overview of the tools in ascending chronological order of their release date and will then provide a comparison and assessment based on basic properties as well as further information on the functionalities provided.

### Overview

#### μ-Argus

μ-Argus is a well-known and early anonymization tool, originating from statistical disclosure control (SDC) research, which was originally published in 1998 by the Centraal Bureau voor de Statistiek, the Netherlands [27]. Argus stands for ‘Anti Re-Identification General Utility System’, μ stands for ‘Microdata’, i.e. individual-level tabular data [28]. The tool provides a relatively low degree of automatization, and it requires users to iteratively transform datasets while measuring reductions in risks and utility. For this, an interactive graphical user interface is implemented. Input can be provided in the form of comma-separated-value (CSV) or fixed-width columnar files as well as SPSS files. The tool is published as an open-source software running on all major operating systems (Windows, Linux, MacOS) and is still maintained today, with the last update at the time of writing in 2021.

#### sdcMicro

sdcMicro is another well-known tool from the statistical domain, originally published in 2007 by Statistics Austria [29]. The tool supports wide range of anonymization methods in the form of a toolbox that is provided as a package for the R statistical computing environment. Analogously to μ-Argus, these different methods can be applied in an iterative manner through a mostly manual process involving repeated evaluations of a dataset’s utility. Through the integration into R, a wide range of statistical methods can be used for this purpose. In addition, the software also features a cross-platform graphical user interface [30], which guides users through the application of the methods supported and produces a log-file in which all operations performed are documented in R code for reproducibility. sdcMicro also features an automated algorithm based on k-anonymity and cell suppression (i.e. the removal of individual cell values). The tool is published as open-source software running on all major operating systems (Windows, Linux, MacOS) and is still maintained today (last update at the time of writing in 2021) [31].

#### OpenAnonymizer

OpenAnonymizer is also a tool developed by computer science researchers from the University of Vienna, Austria, in 2008 [32]. The software is able to automatically anonymize data according to user-selected privacy models and risk thresholds, which can be controlled through a web-based graphical user interface. In contrast to tools from the statistical context, this tool and other tools from the computer science community put a stronger focus on algorithmic approaches to (semi-)automatically transform datasets in such a way that risks measured according to a defined risk or privacy model fall below a user-defined threshold. However, the anonymization algorithm implemented by OpenAnonymizer is quite simple with limited scalability and flexibility. Data and configuration settings need to be provided in an application-specific XML-format. The tool is published as open-source software running on all major operating systems (Windows, Linux, MacOS) but development has ceased with the last update provided at the time of writing in 2009 [33].

#### Cornell anonymization tool

The Cornell anonymization tool (CAT) is another tool from the computer science community, published by Cornell University, USA, in 2009 [34]. CAT combines an automated anonymization process with manual options to adjust output data to reduce residual risks. This process is supported by a comprehensive graphical user interface that can be used to apply transformations to the data and shows, which records in the dataset violate user-defined privacy guarantees as well as how the changes to the dataset have affected basic statistical properties. Data need to be provided in a tool-specific format. The tool is available as open-source software, albeit without a specific license, for Windows operating systems and development has ceased (last update in 2014) [35].

#### TIAMAT

TIAMAT, which stands for ‘Tool for Interactive Analysis of Microdata Anonymization Techniques’, is another software developed by computer science researchers, in this case from Purdue University, USA, in 2009 [36]. It supports different anonymization algorithms, such as Mondrian [37] and k-Member [38] as well as multiple models for analyzing and optimizing the utility of output data, as well as three different privacy and risk models. The processes supported are made available through a cross-platform graphical user interface running on all major operating systems, with a focus on comparing the properties of different anonymization techniques. However, the tool is not available for download and its source is not open.

#### UTD anonymization toolbox

The UDT anonymization toolbox is an early software tool developed by the computer science community and originally published by researchers from the University of Dallas, Texas, USA, in 2010 [39]. The software features several algorithms that apply different transformation methods based on different privacy and risk models, including DataFly [40], Mondrian [37] and Incognito [41]. The software requires data to be encoded into a specific input format based on text files. The tool is published as open-source software running on all major operating systems (Windows, Linux, MacOS), but development has ceased with the last update provided at the time of writing in 2012 [42].

#### Anon

Anon is also a tool developed by computer science researchers, in this case from the University of Klagenfurt, Austria, and was originally published in 2012 [25]. The software is able to automatically anonymize data according to user-selected privacy models and risk thresholds. Analogously to OpenAnonymizer, the anonymization algorithm implemented is quite simple with limited scalability and flexibility. Data and configuration settings need to be provided in an application-specific XML-format. The tool is available as open-source software running on all major operating systems (Windows, Linux, MacOS) but with an unspecified license. The software is not maintained anymore (last update in 2013) [43].

#### ARX data anonymization tool

The ARX data anonymization tool is developed (by the authors of this paper) at the Berlin Institute of Health @ Charité—Universitätsmedizin Berlin, Germany, and was first published in 2012 [13]. ARX also focuses on a high degree of automatization and on flexibility. It supports a wide range of privacy/risk models, transformation models and utility models that can be combined almost arbitrarily. This flexibility is achieved by a generic core algorithm combined with a flexible runtime environment tailored towards anonymization tasks that has been adopted by several other systems, such as Amnesia or SAP HANA Data Anonymization [44]. ARX is available as open-source software running on all major operating systems and is still under active development (last update provided at the time of writing in 2022) [45].

#### SECRETA

SECRETA is a system developed by researchers from the University of Peloponnes, Greece, and published in 2013 [46], which is focused on analyzing the effectiveness and efficiency of anonymization algorithms for tabular as well as set-valued data. It implements nine different algorithms, including four for tabular, data many of which are also supported by other anonymization tools, as well as five different algorithms for set-valued data. It features a cross-platform graphical user interface providing two modes: evaluation and comparison. Input data have to be provided as CSV files. However, analogously to TIAMAT, the tool and its source code are unfortunately not available to the community [47].

#### Probabilistic anonymization

Probabilistic anonymization is a tool developed by researchers from the University of Cyprus, Cyprus and Newcastle University, UK and was published in 2018 [48]. The software differs from the other tools described in this paper by not basing its approach directly on privacy and utility models but by perturbing data through the addition of random noise. Specifically, normally distributed random noise with user-specified variances is added to prevent linkage with other data. Analogously to sdcMicro, the software is provided as a package for the R statistics programming environment and can hence be used with data provided in a range of formats. Probabilistic anonymization has not been updated since its initial release and is distributed without licensing information [49].

#### μ-Ant

μ-Ant is a tool developed at the Center for Cybersecurity Research of Catalonia, Spain, and was first published in 2017 [50]. μ-Ant focuses on microaggregation as a transformation method and puts a special focus on nominal data, implementing semantic mechanisms based on models of the domains of nominal attributes specified using the Web Ontology Language (OWL). In additional to nominal variables, continuous or discrete numerical attributes, dates or basic categorical attributes are supported. Input needs to be provided as CSV files. μ-Ant is a standalone open-source tool implemented in Java, running on all major operating systems. It is still maintained to date, with the last update at the time of writing in 2019. It distributed under the MIT license [51].

#### Amnesia

Amnesia is also a tool developed by computer science researchers, in this case from the University of Thessaly, Greece, and it was first published in 2019 [52]. The software supports a semi-automated anonymization process for tabular and set-valued data (as well as combinations), analogously to SECRETA. For tabular data, it implements a mechanism for classifying different transformations based on the protection provided from re-identification, but it does not support automatically estimating and optimizing the utility of output data. Parts of its codebase are based on ARX. Data can be provided in CSV format and a web-based graphical user interface guides users through the anonymization process. The tool is available as open-source software running on all major operating systems (Windows, Linux, MacOS) and is still under active development (last update in 2022) [53].

#### PrioPrivacy

PrioPrivacy has been developed by the Research Studio Data Science in Vienna, Austria, and was originally published in 2019 [54]. It is a tool for anonymizing tabular data, which intends to provide high flexibility in the choice of variables that need to be protected and the specification of their relevance for the utility of the resulting anonymized data. On the implementation level, it is an extension of the ARX Data Anonymization Tool with additional features—and also restrictions. As it is based on ARX, PrioPrivacy is also implemented in Java and hence compatible with all major platforms and the code is available to the public. It is under active development, with the last update provided in 2021. No information could be found on the license of the software [55].

### Comparison and assessment

An overview of general properties of the tools identified is provided in Table 2.

**Table 2:** Basic properties of the tools identified

Tool	Institution	Country	Language(s)	Release	Last update	License
μ-Argus	Centraal Bureau voor de Statistiek	Netherlands	C++, Java	1998	2021	EUPL
sdcMicro	Statistics Austria	Austria	R	2007	2021	GPL 2
Open Anonymizer	University of Vienna	Austria	Java	2008	2009	Unknown
CAT	Cornell University	USA	C++	2009	2014	Unknown
Tiamat	Purdue University	USA	Java	2009	Unknown	Unknown
UTD	The University of Dallas	USA	Java	2010	2012	GPL 2
Anon	University of Klagenfurth	Austria	Java	2012	Unknown	Unknown
ARX	BIH@Charité	Germany	Java	2012	2022	Apache 2
SECRETA	University of Peloponnes	Greece	C++, Qt	2013	Unknown	Unknown
Probabilistic Anonymization	University of Cyprus, Cyprus and Newcastle University, UK	Greece/UK	R	2018	2018	Unknown
μ-Ant	Center for Cybersecurity Research of Catalonia	Spain	Java	2019	2019	MIT
Amnesia	University of Thessaly	Greece	Java, JavaSript	2019	2022	BSD 3-Clause
PrioPrivacy	Research Studio Data Science	Austria	Java	2019	2021	Unknown

As can be seen, most of the tools were initially released around 2010, with the exception of Probabilistic Anonymization, μ-Ant, Amnesia and PrioPrivacy, which are relatively recent developments (published 2018 and 2019). Nine out of the thirteen tools identified are (at least partly) implemented in Java. The remaining four are implemented in C++ and R. Seven out of thirteen tools have not been released to the public or are released under an unspecified license. Regarding the countries of origin of the primary authors, four of the tools are from Austria, three from the United States, two from Greece, one from Germany, one from Spain, one from the Netherlands and one from Cyprus and the United Kingdom. Only six of the tools are still actively maintained today: Amnesia, ARX, sdcMicro, μ-Argus, μ-Ant and PrioPrivacy. It has to be noted that most of the tools identified must be considered research prototypes and, according to the papers describing them (see prior sections), only μ-Argus, sdcMicro and ARX are mature tools that have been successfully used in a range of real-world applications.

#### Transformation models

Data transformation methods can be classified into truthful and non-truthful approaches (see [56] for an overview). Well-known truthful methods include generalization, where values are replaced by semantically consistent values taken from a user-defined hierarchical representation of the domain of values. Generalization hierarchies can also be represented as functions, which can be used to perform on-the-fly categorization of continuous attributes during anonymization. A top code for a variable is an upper bound for all values of that variable in a dataset. Similarly, a bottom code is a lower bound for all values of a variable in a dataset. Top- and bottom coding can be implemented by using hierarchies that truncate values exceeding a user-specified range. Generalization can be applied in different ways. With full-domain generalization, all values of an attribute are transformed to the same generalization level in all records. With multi-dimensional generalization, different generalization schemes can be applied to different subsets of the records, but the same records will always be generalized in the same manner. With local generalization, this guarantee is not provided. Suppression is another truthful method implementing the removal of data, which can be performed on cell, attribute and record level. Important non-truthful transformation methods include the addition of noise and microaggregation, which means that groups of values are replaced with aggregates, such as the arithmetic or geometric mean or an interval covering their range.

As mentioned, we will put a specific focus in our analysis on methods that are supported in (semi-) automated anonymization processes by the tools identified. μ-Argus supports generalization and suppression for anonymizing combinations of up to three key variables/quasi-identifiers. sdcMicro supports enforcing one privacy model (k-anonymity) using cell suppression. The UTD anonymization toolbox implements full-domain and multi-dimensional generalization, bottom-coding, record, cell and attribute suppression. CAT supports full-domain, record and attribute suppression as well as top- and bottom-coding. Amnesia implements full-domain and local generalization as well as top- and bottom-coding. TIAMAT supports local generalization, cell suppression and microaggregation. OpenAnonymizer and Anon both support full-domain generalization, top- and bottom coding as well as attribute suppression. SECRETA implements local generalization and cell suppression. μ-Ant supports microaggregation through clustering and then replacing values by cluster averages and PrioPrivacy provides generalization in combination with suppression, as local recoding. Probabilistic Anonymization is based on noise addition. Finally, ARX implements full-domain and multi-dimensional generalization, top- and bottom coding, categorization, cell-, attribute- and record-suppression, sampling and microaggregation.

In addition to automated transformation, several tools also support manual modifications to input data. Examples include local suppression, randomization, noise addition and microaggregation in μ-Argus as well as sampling, microaggregation, noise addition and rank swapping in sdcMicro.

#### Utility models

A wide variety of utility models has been proposed in the literature (see [18] for an overview). Many of the tools provide only limited support of methods for optimizing the utility of output data automatically during the anonymization process. SECRETA implements several such features with a specific focus on transaction data. Amnesia will not automatically optimize the utility of output data. OpenAnonymizer, Anon, the UTD Anonymization Toolbox and sdcMicro only support a model minimizing the degree of generalization applied. In contrast, ARX and Tiamat support several more complex optimization functions, such as normalized certainty penalty, loss or the classification metric for tailoring output towards machine learning purposes. μ-Ant calculates the sum of squared error and supports comparisons of variances and means between input and output data. As PrioPrivacy is based on ARX it supports all utility models provided by the software but puts a specific focus on Non-Uniform Entropy. Several tools, including ARX, sdcMicro, CAT, μ-Argus, SECRETA and Amnesia, also provide features for illustrating and analyzing the utility of output data, e.g. by means of contingency tables or density graphs as well as further types of statistical methods. Probabilistic Anonymization does not support any specific methods for utility evaluation.

#### Privacy models

As already mentioned above, a wide variety of privacy models has been proposed in the literature [22]. These address different privacy threats. Membership disclosure means that an adversary, i.e. a malicious entity trying to breach the privacy of individuals, is able to determine whether or not data about an individual is contained in a dataset. While this does not directly disclose information from the dataset itself, it may allow an adversary to infer meta-information, e.g. if the data are from a disease-specific registry. Attribute disclosure can be achieved without linking a person to a specific element in a dataset. It occurs when sensitive attributes from the dataset can be associated to individuals. Identity disclosure (or re-identification) means that an individual can be linked to a particular record.

**Figure 2:**
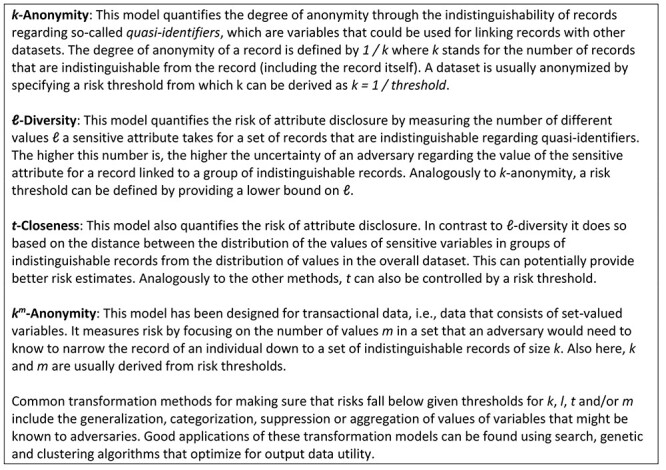
Info box providing a brief explanation of the privacy models listed in Table 3.

An overview of the models that can be enforced automatically by at least two of the tools identified is listed in Table 3. ℓ-Diversity [57] and t-closeness [58] protect data from attribute disclosure, while k-anonymity [14] as well as k^m^-anonymity [59] protect data from identity disclosure. A brief explanation of the different models is provided in Figure 2.

**Table 3:** Privacy models supported by at least two of the tools identified

Privacy model	Model type	μ-Argus	sdcMicro	Open anonymizer	CAT	TIAMAT	UTD	Anon	ARX	SECRETA	μ-Ant	Amnesia	Prioprivacy	Probabilistic anonymization
*k* ^m^-Anonymity	Syntactic/statistical									X		X		
*k*-Anonymity	Syntactic/statistical	X^a^	X	X		X	X	X	X	X	X	X	X	
*ℓ*-Diversity	Syntactic/statistical				X		X	X	X					
*t*-Closeness	Syntactic/statistical				X		X		X		X			

^a^Only for combinations of one, two or three key variables

As can be seen, only amnesia and SECRETA support k^m^-anonymity, which is a privacy model transferring the concept of k-anonymity to set-valued data (e.g. sets of diagnoses of patients). k-Anonymity, which is the most well-known and common model, is supported by all tools analyzed, apart from CAT. We note that μ-Argus supports k-anonymity only for up to three key variables. l-Diversity and t-closeness are supported by four and three of the tools, respectively. Probabilistic anonymization does not implement any of the models listed in Table 3, but provides implicit protection from re-identification achieved by noise addition.

All tools except ARX support automated anonymization exactly for the models listed in Table 3. ARX also supports δ-Presence [60], which protects data from membership disclosure, advanced methods for protecting data from identity disclosure (average risk [61], k-map [14], population uniqueness [62], game-theoretic models [16]), additional models protecting data from attribute disclosure (δ-disclosure privacy [63] and β-likeness [64]) as well as differential privacy [17], which is a stringent model protecting output data from all types of disclosure mentioned.

#### Performance

Some anonymization problems have been shown to be NP-hard [65], and optimal solutions can therefore often only be computed for relatively small problem sizes (up to about 15 quasi-identifiers). However, a range of heuristic and approximate algorithms have been developed which can also provide good solutions for larger problem sizes (see, e.g. [66]). The performance of an anonymization algorithm will usually strongly depend on the statistical properties of a real-world dataset. We deliberately decided to not include a performance comparison in this review for the following reasons:

(i) As mentioned previously, it can be difficult to compare anonymization tools to each other without comparing apples to oranges due to the differences in their feature sets.
(ii) A wide range of papers have already studied the performance of most of the anonymization tools covered in this paper.

In regards to (ii), careful performance comparisons between ARX, the UTD anonymization toolbox and μ-ANT as well between ARX and sdcMicro have been presented in [13, 67]. A comparison between ARX and Amnesia has been published recently [68]. The performance of ANON has been studied in [25]. From the results of these papers, it can be concluded that ARX outperforms the other anonymization tools studied. PrioPrivacy and Amnesia are based on the code of ARX and will hence provide comparable performance. The algorithm implemented by probabilistic anonymization is trivial in terms of runtime and memory complexity and will hence execute very fast. A performance comparison with μ-Argus would be of little practical relevance, as it can only handle very limited anonymization problems. CAT, TIAMAT and SECRETA are not available for performance evaluations.

We note that the three tools considered particularly relevant for practical applications by the authors (see next section) are all expected to provide reasonable performance in real-world settings. This means that the anonymization of a dataset with 10 key variables that could be used for re-identifying individuals (usually an exponential factor) takes up to about 1 s for 10 000 records (usually a linear factor), 10 s for 100 000 records and 100 s for 1 000 000 records (see [13, 67] for detailed experimental analyses). We note that the tools will typically just use one CPU core per anonymization process (Amnesia also supports a parallelized k-anonymity algorithm.) If execution times in this order of magnitude are not acceptable or the dataset to be anonymized is much larger, some tools support additional heuristics that can be used to reduce computational complexity or data can also be split into different chunks, which can then be processed independently and also in parallel (see [67] for an example using ARX). Both methods of increasing performance, however, can come with a reduction of output data utility.

## Discussion

### Principal results

Based on the results of our analyses, we consider three anonymization tools to be particularly relevant for most use cases: (i) ARX can serve as a scalable basis for implementing anonymization pipelines supporting a wide range of anonymization, risk assessment and risk management methods for tabular data, (ii) amnesia is particularly relevant for integrating methods tailored towards set-valued data, e.g. sets of diagnosis codes, into anonymization processes, and (iii) sdcMicro provides a powerful environment for anonymizing data using a semi-automated process within the statistical computing environment R. The other tools identified are mostly research prototypes that are either not publicly available, not sufficiently scalable, not maintained anymore or lack critical functionalities. The information provided in this article can support users with the selection of an appropriate tool, e.g. based on the privacy models supported.

### Limitations and related work

In this article, we have focused on anonymization tools for tabular data. However, other types of data are also relevant for biomedical research, in particular clinical texts, medical images and omics data. Anonymization methods for clinical texts (e.g. physician letters or progress notes in electronic health records) typically follow a rule-based approach, e.g. Scrubber from the US National Library of Medicine [69], or implement machine learning-based methods (see [70] for a recent study) to remove identifying tokens from texts or replace them with consistent synthetic data [71]. Software for anonymizing medical images also aims to remove identifying characteristics. This is particularly important for images of the head, for example, which is why a number of software solutions have been developed for removing facial features from medical resonance imaging (MRI) images (defacing; see [72] for an overview). There are also proposals for anonymization methods applicable to the field of genomic data [73], although interactive anonymization and interfaces for availability requests are of particular relevance in this context (see [74] for a recent review). Tools have also been proposed focusing on data represented in common interoperability standards, such as clinical data in the HL7 FHIR format (see, e.g. [75]). Another limitation of our work is the focus on articles published in English.

As an alternative or addition to anonymization, the generation of synthetic data, especially by means of Generative Adversarial Networks (GANs), is increasingly being discussed. However, whether these methods are really superior to classic anonymization methods is subject of ongoing research (see, e.g. [76]). In future work, we plan to also perform structured reviews of open tools for protecting further types of data.

## Conclusion

In this article, we have provided a systematic review of open anonymization tools for tabular phenotype data. We have shown that the landscape consists mainly of research prototypes and that only a handful of mature tools are available. We recommend that researchers searching for anonymization tools for practical applications take a closer look at ARX for automated anonymization of relational data, Amnesia for automated anonymization of set-valued data, and sdcMicro as a library and tool for mostly manual anonymization processes.

**Key Points**

- Data anonymization is complex and providing transparency on the strengths and weaknesses of publicly available tools for tabular data is challenging.
- We performed a systematic review of open anonymization tools for structured phenotype data described in the literature.
- We derive recommendations for tools to use for anonymizing phenotype datasets with different properties and in different contexts.

## Supplementary Material

Paper-Tool-Review-R1-Supplement-1_bbac440

## Data Availability

The data underlying this article are available in the article and in its online Supplementary Material.

## Author Biographies

**Anna C. Haber** holds a Diploma in Mathematics and is a PhD student at the Medical Informatics Group in the Center of Health Data Science at the Berlin Institute of Health at Charité—Universitätsmedizin Berlin.

**Ulrich Sax** is a professor of Medical Informatics and Head of Research Group Infrastructure for Translational Research in the Department of Medical Informatics at the University Medical Center Göttingen.

**Fabian Prasser** is a professor of Medical Informatics and Head of the Medical Informatics Group in the Center of Health Data Science at the Berlin Institute of Health at Charité—Universitätsmedizin Berlin.

## References

[1] Aronson SJ , Rehm HL. Building the foundation for genomics in precision medicine. Nature 2015;526:336–42. 26469044 10.1038/nature15816 PMC5669797

[2] Malone ER , Oliva M, Sabatini PJB, et al. Molecular profiling for precision cancer therapies. Genome Med 2020;12:8. 31937368 10.1186/s13073-019-0703-1 PMC6961404

[3] Collins FS , Varmus H. A new initiative on precision medicine. N Engl J Med 2015;372:793–5. 25635347 10.1056/NEJMp1500523 PMC5101938

[4] Cyranoski D . China embraces precision medicine on a massive scale. Nature 2016;529:9–10. 26738574 10.1038/529009a

[5] Gefenas E , Lekstutiene J, Lukaseviciene V, et al. Controversies between regulations of research ethics and protection of personal data: informed consent at a cross-road. Med Health Care Philos 2022;25:23–30. 34787769 10.1007/s11019-021-10060-1 PMC8595272

[6] Wilkinson MD , Dumontier M, IjJ A, et al. The FAIR guiding principles for scientific data management and stewardship. Sci Data 2016;3:160018. 26978244 10.1038/sdata.2016.18 PMC4792175

[7] Holub P , Kohlmayer F, Prasser F, et al. Enhancing *reuse* of data and biological material in medical research: From fair to fair-health. Biopreservation Biobanking 2018;16:97–105. 29359962 10.1089/bio.2017.0110 PMC5906729

[8] United States Congress . Health insurance portability and accountability act of 1996. Public Law 1996;104:191. 16477734

[9] Regulation (EU) 2016/679 of the European Parliament and of the Council of 27 April 2016 on the protection of natural persons with regard to the processing of personal data and on the free movement of such data, and repealing Directive 95/46. Off J Eur Union OJ 2016;59:294.

[10] WMA (World Medical Association) . World Medical Association Declaration of Helsinki: ethical principles for medical research involving human subjects. JAMA 2013;310(20):2191–4. 10.1001/jama.2013.281053. 24141714

[11] Williams G , Pigeot I. Consent and confidentiality in the light of recent demands for data sharing. Biom J 2017;59:240–50. 26841369 10.1002/bimj.201500044

[12] El Emam K , Jonker E, Arbuckle L, et al. A systematic review of re-identification attacks on health data. PLoS ONE 2011;6:e28071. 22164229 10.1371/journal.pone.0028071 PMC3229505

[13] Prasser F , Eicher J, Spengler H, et al. Flexible data anonymization using ARX—Current status and challenges ahead. Softw Pract Exp 2020;50:1277–304.

[14] Sweeney L . k-Anonymity: a model for protecting privacy. Int J Uncertain Fuzziness Knowl-Based Syst 2002;10:557–70.

[15] Hoshino N . Applying Pitman’s sampling formula to microdata disclosure risk assessment. J Off Statn.d.;17:499–520.

[16] Prasser F , Gaupp J, Wan Z, et al. An open source tool for game theoretic health data de-identification. AMIA Annu Symp Proc AMIA Symp 2017;2017:1430–9. 29854212 PMC5977602

[17] Dwork C , Roth A. The algorithmic foundations of differential privacy. Found Trends®. Theor Comput Sci 2013;9:211–407.

[18] Fung BCM (ed). Introduction to Privacy-Preserving Data Publishing: Concepts and Techniques. Boca Raton: Chapman & Hall/CRC, 2011.

[19] Abowd JM . The U.S. Census Bureau Adopts Differential Privacy. In: Proc. 24th ACM SIGKDD Int. Conf. Knowl. Discov. Data Min. London, UK: ACM, 2018, 2867–7.

[20] Oswald M . Anonymisation standard for publishing health and social care data specification (Process Standard). Leeds, UK: Information Standards Board for Health and Social Care, 2013.

[21] 13th National People’s Congress (Standing Committee of the National People’s Congress of the People’s Republic of China). Personal Information Protection Law of the People’s Republic of China, 2021.

[22] Wagner I , Eckhoff D. Technical privacy metrics: a systematic survey. ACM Comput Surv 2018;51(57):1–57 38.

[23] Bild R , Kuhn KA, Prasser F. Better safe than sorry—implementing reliable health data anonymization. Stud Health Technol Inform 2020;270:68–72. 32570348 10.3233/SHTI200124

[24] Page MJ , McKenzie JE, Bossuyt PM, et al. The PRISMA 2020 statement: an updated guideline for reporting systematic reviews. BMJ 2021;372:n71. 33782057 10.1136/bmj.n71 PMC8005924

[25] Ciglic M , Eder J, Koncilia C. ANON—a flexible tool for achieving optimal k-anonymous and l-diverse tables. Klagenfurt, AUT: University of Klagenfurt, 2014, 1–23.

[26] Iyengar VS . Transforming data to satisfy privacy constraints. In: *Proc. Eighth ACM SIGKDD Int. Conf. Knowl. Discov. Data Min.*—*KDD 02, Edmonton*. Alberta, Canada: ACM Press, 2002, 279.

[27] Hundepool A , Domingo-ferrer J, Franconi L, et al. Handbook on Statistical Disclosure Control (Version 1.2). CENEX SDC (Centre of Excellence for Statistical Disclosure Control). 2010, 1–216.

[28] Willenborg L , Hundepool A, Wessels A, et al.. mu-ARGUS User’s Manual (Version 2.5). Voorburg, NL: Centraal Bureau voor de Statistiek (Statistics Netherlands), 1998.

[29] Templ M , Kowarik A, Meindl B. Statistical disclosure control for micro-data using the R package sdcMicro. J Stat Softw 2015;67:1–36.

[30] Meindl B , Templ M. Feedback-based integration of the whole process of data anonymization in a graphical interface. Algorithms 2019;12:191.

[31] sdcTools/sdcMicro: sdcMicro 2021. https://github.com/sdcTools/sdcMicro (17 December 2021, date last accessed).

[32] Stark K . Scientific Workflows, Data Provenance Management and Data Anonymization in Context of the Genome Austria Tissue Bank. Vienna: Universität Wien, 2013.

[33] Open Anonymizer download | SourceForge.net . https://sourceforge.net/projects/openanonymizer/(17 December 2021, date last accessed).

[34] Xiao X , Wang G, Gehrke J. Interactive anonymization of sensitive data. In: Proc. 2009 ACM SIGMOD Int. Conf. Manag. Data. New York, NY, USA: Association for Computing Machinery, 2009, 1051–4.

[35] Download Cornell Anonymization Toolkit from SourceForge.net . https://sourceforge.net/projects/anony-toolkit/files/Documents/cat-mannual-1.0.PDF/download (17 December 2021, date last accessed).

[36] Dai C , Ghinita G, Bertino E, et al. TIAMAT: a tool for interactive analysis of microdata anonymization techniques. PVLDB 2009;2:1618–21.

[37] LeFevre K , DeWitt DJ, Ramakrishnan R. Mondrian multidimensional k-anonymity. In: 22nd Int. Conf. Data Eng. (ICDE’06). Atlanta, GA, USA: IEEE, 2006, 1–25.

[38] Byun J-W , Kamra A, Bertino E, et al. Efficient k-anonymization using clustering. Dent Tech 2007;4443:188–200.

[39] Kantarcioglu M , Inan A, Kuzu M. *UT Dallas Anonymization Toolbox*—*Manual*. anonManual.pdf. http://cs.utdallas.edu/dspl/cgi-bin/toolbox/anonManual.pdf (27.09.2022, date last accessed)

[40] Sweeney L . Datafly: a system for providing anonymity in medical data. In: Lin TY, Qian S (eds). Database Secur. XI Status Prospects. Boston, MA: Springer US, 1998, 356–81.

[41] LeFevre K , Dewitt DJ, Ramakrishnan R. Incognito: efficient full-domain K-anonymity. In: Proc. 2005 ACM SIGMOD Int. Conf. Manag. Data. New York, NY, USA: Association for Computing Machinery, 2005, 49–60.

[42] UTD Anonymization ToolBox . http://cs.utdallas.edu/dspl/cgi-bin/toolbox/(27 September 2021, date last accessed).

[43] ANON 2013. https://www.tmf-ev.de/Produkte/Uebersicht/ctl/ArticleView/mid/807/articleId/1290/P100201.aspx.

[44] Kessler S , Hoff J, Freytag J-C. SAP HANA goes private: from privacy research to privacy aware enterprise analytics. Proc VLDB Endow 2019;12:1998–2009.

[45] *ARX*—*Data Anonymization Tool | A Comprehensive Software for Privacy-Preserving Microdata Publishing*. https://arx.deidentifier.org/(15 December 2021, date last accessed).

[46] Poulis G , Gkoulalas-Divanis A, Loukides G, et al. SECRETA: A System for Evaluating and Comparing RElational and Transaction Anonymization Algorithms. Konstanz: OpenProceedings.org, University of Konstanz, 2014, 620–3.

[47] The SECRETA system . http://users.uop.gr/∼poulis/SECRETA/(17 December 2021, date last accessed).

[48] Avraam D , Boyd A, Goldstein H, et al. A software package for the application of probabilistic anonymisation to sensitive individual-level data: a proof of principle with an example from the ALSPAC birth cohort study. Longitud Life Course Stud 2018;9:433–46.

[49] Probabilistic Anonymisation . davraam/Probabilistic_Anonymisation: R functions for (a) applying probabilistic anonymisation on individual-level data and (b) calculating a re-identification risk measure. 2018. https://github.com/davraam/Probabilistic_Anonymisation (27 September 2022, date last accessed).

[50] Sánchez D , Martínez S, et al. μ -ANT: semantic microaggregation-based anonymization tool. Bioinforma Oxf Engl 2020;36:1652–3. 10.1093/bioinformatics/btz792 31621826

[51] CrisesUrv/microaggregation-based_anonymization_tool: Microaggregation-based Anonymization Tool is a tool to protect datasets applying microaggregation algorithms in order to fulfill k-anonymity or k-anonymity and t-closeness. 2019. https://github.com/CrisesUrv/microaggregation-based_anonymization_tool (27 September 2022, date last accessed).

[52] Dimakopoulos MT Dimitris Tsitsigkos and Nikolaos. *Amnesia Anonymization Tool*—*Data Anonymization Made Easy*. https://amnesia.openaire.eu/ (17 December 2021, date last accessed).

[53] dTsitsigkos/Amnesia 2022. https://github.com/dTsitsigkos/Amnesia (27 September 2022, date last accessed).

[54] Bampoulidis A , Markopoulos I, Lupu M. PrioPrivacy: a local recoding k-anonymity tool for prioritised quasi-identifiers. In: *IEEEWICACM Int. Conf. Web Intell.*—*Companion*. New York, NY, USA: Association for Computing Machinery, 2019, 314–7.

[55] alex-bampoulidis/prioprivacy 2021. https://github.com/alex-bampoulidis/prioprivacy (27 September 2022, date last accessed).

[56] Templ M . Statistical Disclosure Control for Microdata. Cham: Springer International Publishing, 2017.

[57] Machanavajjhala A , Gehrke J, Kifer D, et al. L-diversity: privacy beyond k-anonymity. In: 22nd Int. Conf. Data Eng. ICDE06. Atlanta, GA, USA: IEEE, 2006, 24–4.

[58] Li N , Li T, Venkatasubramanian S. t-Closeness: privacy beyond k-anonymity and l-diversity. In: 23rd Int. Conf. Data Eng. (ICDE’07). Istanbul, Turkey, USA: IEEE, 2007, 106–15.

[59] Terrovitis M , Mamoulis N, Kalnis P. Privacy-preserving anonymization of set-valued data. Proc VLDB Endow 2008;1:115–25.

[60] Nergiz ME , Atzori M, Clifton C. Hiding the presence of individuals from shared databases. In: Proc. 2007 ACM SIGMOD Int. Conf. Manag. Data. New York, NY, USA: Association for Computing Machinery, 2007, 665–76.

[61] Prasser F , Kohlmayer F, Kuhn KA. The importance of context: risk-based de-identification of biomedical data. Methods Inf Med 2016;55:347–55. 27322502 10.3414/ME16-01-0012

[62] Dankar FK , Emam KE, Neisa A, et al. Estimating the re-identification risk of clinical data sets. BMC Med Inform Decis Mak 2012;12:66. 22776564 10.1186/1472-6947-12-66 PMC3583146

[63] Brickell J , Shmatikov V. The cost of privacy: destruction of data-mining utility in anonymized data publishing. In: *Proceeding 14th ACM SIGKDD Int. Conf. Knowl. Discov. Data Min.*—*KDD 08*. Las Vegas, Nevada, USA: ACM Press, 2008, 70.

[64] Cao J , Karras P. Publishing microdata with a robust privacy guarantee. ArXiv12080220 Cs 2012;5:1388–99.

[65] Meyerson A , Williams R. On the complexity of optimal K-anonymity. In: *Proc. Twenty-Third ACM SIGMOD-SIGACT-SIGART Symp. Princ. Database Syst.*—*PODS 04*. Paris, France: ACM Press, 2004, 223.

[66] Meurers T , Bild R, Do K-M, et al. A scalable software solution for anonymizing high-dimensional biomedical data. GigaScience 2021;10:giab068. 34605868 10.1093/gigascience/giab068 PMC8489190

[67] Prasser F , Spengler H, Bild R, et al. Privacy-enhancing ETL-processes for biomedical data. Int J Med Inf 2019;126:72–81. 10.1016/j.ijmedinf.2019.03.006 31029266

[68] Tomás J , Rasteiro D, Bernardino J. Data anonymization: an experimental evaluation using open-source tools. Future Internet 2022;14:167.

[69] US National Library of Medicine. NLM Scrubber 2022. https://scrubber.nlm.nih.gov/ (27 September 2022, date last accessed).

[70] Yang X , Lyu T, Li Q, et al. A study of deep learning methods for de-identification of clinical notes in cross-institute settings. BMC Med Inform Decis Mak 2019;19:232. 31801524 10.1186/s12911-019-0935-4 PMC6894104

[71] Carrell D , Malin B, Aberdeen J, et al. Hiding in plain sight: use of realistic surrogates to reduce exposure of protected health information in clinical text. J Am Med Inform Assoc JAMIA 2013;20:342–8. 22771529 10.1136/amiajnl-2012-001034 PMC3638183

[72] Theyers AE , Zamyadi M, O’Reilly M, et al. Multisite comparison of MRI defacing software across multiple cohorts. Front Psych 2021;12:617997. 10.3389/fpsyt.2021.617997 PMC7943842 33716819

[73] Wan Z , Vorobeychik Y, Xia W, et al. Expanding access to large-scale genomic data while promoting privacy: a game theoretic approach. Am J Hum Genet 2017;100:316–22. 28065469 10.1016/j.ajhg.2016.12.002 PMC5294764

[74] Wan Z , Hazel JW, Clayton EW, et al. Sociotechnical safeguards for genomic data privacy. Nat Rev Genet 2022;23:429–45. 10.1038/s41576-022-00455-y PMC8896074 35246669

[75] fair4health/data-privacy-tool: . FAIR4Health Data Privacy Tool, 2022. https://github.com/fair4health/data-privacy-tool (27 September 2022, date last accessed).

[76] Stadler T , Oprisanu B, Troncoso C. Synthetic data–anonymisation groundhog day. In 31st USENIX Security Symposium (USENIX Security 22). Boston, MA, USA: Usenix Association, 2020, 1451–68.

